# Supplementary material for: Cross-partisan discussions reduced political polarization between UK voters, but less so when they disagreed
Source: Commun Psychol. 2024 Jan 5;2:5. doi: 10.1038/s44271-023-00051-8 (PMC11332051; doi:10.1038/s44271-023-00051-8)
Supplement: Supplementary file 1 — Supplementary Information [file 44271_2023_51_MOESM1_ESM.pdf]

Supplementary Information (SI) Appendix for Cross-partisan discussions reduced political polarization between UK voters, but less so when they disagreed

Jona de Jong*

2023-12-07

Contents

A Supplementary Notes 1. Additional evidence from real-world cross-partisan discussions in the Netherlands **3**
- A.1 Set-up and identification . . . 3
- A.2 Results . . . 4

B Supplementary Notes 2. Recruitment and final sample **5**
- B.1 Recruitment and exclusion . . . 5
- B.2 Attrition . . . 8
- B.3 Balance table, manipulation check . . . 10
- B.4 All survey questions . . . 11

C Supplementary analysis **16**
- C.1 Supplementary Methods 1. Affective polarization (Fig. 3 main paper) . . . 16
  - C.1.1 Regression table Fig. 3 . . . 16
  - C.1.2 Within-individual effects of the discussion on sympathy, friends and discussion . . . 18
  - C.1.3 Placebo test . . . 18
  - C.1.4 Results by party . . . 19
- C.2 Supplementary Methods 2. Discussion effects by issue disagreement (Fig. 4 main paper) . . . 21
  - C.2.1 Randomization check . . . 21
  - C.2.2 Regression tables and interaction models figure 4 . . . 21
- C.3 Supplementary Methods 3. Opinion change (Fig. 5 main paper) . . . 27
  - C.3.1 Baseline opinions, and opinions before the discussion, by treatment assignment . . . 27
  - C.3.2 Models figure 5 main paper . . . 28

*European University Institute, Email: jona.dejong@eui.eu

C.3.3 Conservatives hide their views when about to discuss immigration . . 32
C.3.4 Robustness figure 5: within-results 34
C.3.5 Robustness: view change between pre-screener and follow-up survey . 35
C.4 Supplementary Methods 4. Mechanisms 40
C.4.1 Presence of mechanisms not correlated with treatment assignment . . 40
C.4.2 Expressed disagreement only predicted by issue disagreement. Levels of disagreement balanced across treatment and control 41
C.4.3 Regression models for figure 6 main paper 43
C.4.4 Regression models for figure 7 main paper 44
C.5 Supplementary Methods 5. Further pre-registered hypotheses and deviations from the pre-analysis plan 46
C.5.1 The moderating effect of out-party closeness and ideology 46
C.5.2 Political tolerance 47
C.5.3 Topic 48

A Supplementary Notes 1. Additional evidence from real-world cross-partisan discussions in the Netherlands

In the five weeks running up to the March 2021 Dutch Parliamentary elections, Civinc and I made the platform publicly available in Dutch society.[1] The largest vote advice application in the Netherlands advertised the platform on their website, and the initiative frequently appeared in various national media outlets.[2]

The initiative was not set up as a research study and most data was not saved. The NGO asked for informed consent after the discussion, to not give people the feeling that they were participating in a study. To arrive at the informed consent page, participants were supposed to click on a small button titled 'stop/to results'. Unfortunately, as pre-testing revealed, the great majority of participants overlooked this button and simply closed their browser. Their data was immediately deleted, yielding a final N of about 4000 respondents. Analysis of this data hinges on the assumption that giving informed consent is not affected by the discussion, which I cannot test. Thus, this study should mainly be seen as proof of scalability, and complementary suggestive evidence that real-world discussions can also reduce polarization.

A.1 Set-up and identification

The platform that we made publicly available is an earlier version of the one used for the controlled experiment in the main paper. When participants entered, they were asked to choose one of twelve parties. Each party was classified as belonging to one ideological group: left, center-left, center, center-right and right. The algorithm then matched participants based on maximum ideological distance. During the discussion, participants could change statements and discussion partners as often as they liked. A bot gave discussion tips.

The few participants who arrived at the final screen were asked three questions. The first is my main outcome measure. It asks: 'Insofar as you identify with 'left' or 'right', how sympathetic do you find people from the other side of the political spectrum' (0-10). It fits the fact that in the Netherlands, voters often switch between parties, but not between blocks of parties. Thus, many do not have strong party identities, but do have clear block identities. The second question asks whether they enjoyed the discussion. The third is an open-ended question asking participants how they found their experience.

My identification strategy uses the fact that participants were matched live, and often could not find a match. I use the open-response question to identify participants who attempted to have a discussion, could not find a match, and explicitly told us that this happened. I thus compare outcomes of participants who had a discussion to outcomes of

[1]The study received ethics approval by the ethics board of the European University Institute on the 25th of March 2021 and was pre-registered at osf. I received the data on the 7th of April 2021 - https://osf.io/y9zr7/

[2]See www.stemwijzer.nl for the VAA, and this Youtube channel for media appearances (https://www.youtube.com/channel/UCjMjDukf6XHF9pkW0hKAcpg)

Supplementary Table 1: Sympathy by discussion condition

	Sympathy by discussion condition
Intercept	6.407^{***}
	(0.369)
Negative discussion	-2.142^{***}
	(0.356)
Negative, non-response	-2.068^{***}
	(0.363)
Non-response	-0.902^{**}
	(0.279)
R^2	0.176
Adj. R^2	0.098
Num. obs.	983

$^{***}p < 0.001$; $^{**}p < 0.01$; $^{*}p < 0.05$

participants who could not find a match, or ran into an unresponsive partner. In a way, these participants remain untreated. I further sub-categorize this group into participants who conveyed anger or disappointment with not being able to have a discussion, and blaming it on their discussion partner. These participants were essentially also treated, just with a different treatment of feeling ignored by an out-partisan. Others expressed themselves in more neutral terms (e.g. 'too bad', 'nobody replied', 'there was nobody here') and did not blame this on their discussion partner. This group of 78 participants is the closest this study can get to a control group. These groups do not differ significantly from each other on any of the relevant variables in the data. Using only participants who filled in the open-response item further limits my N to 973 participants.

A.2 Results

Supplementary Fig. 1 shows outgroup sympathy scores for four groups: those who had a discussion ('Discussion' - N = 817), those who ran into an unresponsive partner and did not seem to mind ('Non-response, neutral'/the 'control group' - N = 78), those who ran into an unresponsive partner and found this to be a negative experience ('Non-response, negative' - N = 43) and those who had a discussion, and found this to be a negative experience ('Negative discussion' - N = 45). Table 1 shows results from a model that uses these categories to predict out-partisan sympathy scores after the discussion, controlling for the party they selected and the date and hour on the platform. Results are in line with the findings in the main paper. Those who had a discussion have significantly higher sympathy scores than those in the closest thing to a control group (point estimates show the mean difference between the groups, bars are 95% Confidence Intervals). Those with negative experiences report significantly lower out-partisan affect. This results should be viewed as a first, promising indication that real-world discussions can also reduce polarization.

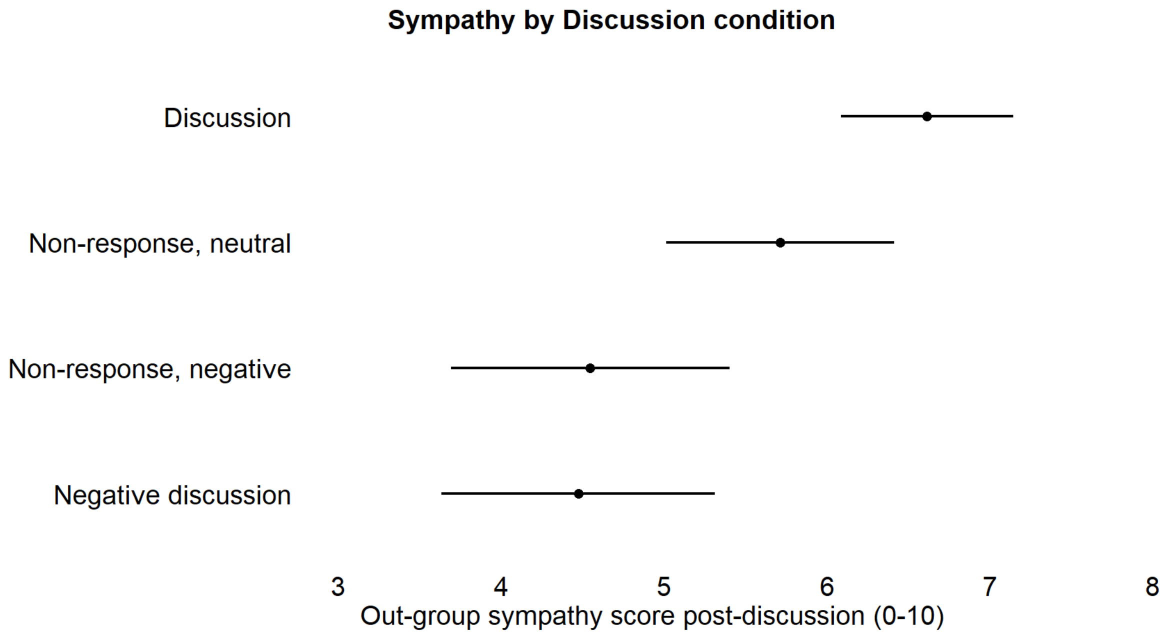

Supplementary Figure 1: Sympathy score by discussion condition. Error bars are 95% CIs.

B Supplementary Notes 2. Recruitment and final sample

B.1 Recruitment and exclusion

I recruited participants through Prolific, a research company. Prolific has several features that makes it useful for conducting studies that require following up with participants, such as extensive pre-screening options, direct communication with respondents and a large and active UK user base. I surveyed participants three times: a few days before the experiment, right after the experiment and two-three weeks after the experiment. Participants took the pre-screener and follow-up survey in Qualtrics, but answered pre and post-discussion questions on the Civinc platform where they also had the discussion. I recruited them in nine waves, on nine different days in June 2022.

A total of 2279 people took the pre-screener. The survey asked several questions on demographics and politics, and took an average of three minutes to complete. Based on answers to the first wave of the pre-screener, I selected two statements out of five that were to be the discussion topics: immigration and redistribution. Selection was based on highest average disagreement between partisans in the first wave. I limited eligibility for the discussion experiment to those who voted Labour or Conservative in the last election, have the UK nationality, have command of the English language and are willing to return for 'a brief, anonymous discussion on a novel chat platform'. This left 1523 respondents. Each respondent was asked to provide a time slot on which they could return for the experiment. Some did not do this, and I randomly dropped mostly Labour voters to invite a balanced sample of Labour and Conservative voters to come to the platform in each wave. A total of 1090 respondents were invited to take part in the experiment. I sent the invitation through

a private message in Prolific's internal messaging system. Invitees were asked to mark the time slot in their agenda. They were also instructed that participation and payment were conditional upon arriving exactly on time. A few hours before each time slot, I make the study available on Prolific to invited participants. Thirty minutes before each time slot, I sent participants a final message reminding them that the study was about to start.

777 participants, around 60% of those invited, showed up. An algorithm immediately randomly assigned them to the control condition or the treatment condition. About 50 were excluded because they were too late or too early to be matched. Others were excluded, but paid, because they could not find a match. This was due to an imbalance in partisanship within waves. Though invitations to each wave were balanced by partisanship, attendance was not always. As instructed, 78 participants reported to me by private message that they could not find a match, after having waited for at least five minutes. The data show how long participants spend on the survey, and whether they enter the matching process, allowing me to see whether they actually waited five minutes. Some participants did not contact me, but instead re-entered the platform. In this case, I took out both these participants and their discussion partner. A few others reported a technical glitch: the platform froze, or they were thrown out. If this happened before the treatment, I took them out. If it happened during the treatment, I also took out their discussion partner. I left them in if it happened after the treatment. Supplementary Fig. 2 shows that only education levels are predictive of whether participants made it into the final sample or not. Those in the final sample are more likely to have a master's degree and those who are not in the final sample are more likely to have only higher secondary education. Other than this, there is a balance on treatment assignment as well as policy attitudes, closeness towards parties, and ideology in the pre-screener.

Include_in_analysis?		FALSE			TRUE		
Variable	N	Mean	SD	N	Mean	SD	Test
Treatment	160			595			X2=0.088
... Control	79	49%		304	51%		
... Treatment	81	51%		291	49%		
t0_gender	158			591			X2=1.641
... Female	81	51%		339	57%		
... Male	77	49%		252	43%		
education	158			591			X2=18.574***
... Higher secondary	34	22%		171	29%		
... Bachelor's degree	64	41%		239	40%		
... Lower	13	8%		38	6%		
... Lower secondary	17	11%		23	4%		
... Higher national certificate, NVQ level 4	11	7%		23	4%		
... Master/PhD	19	12%		95	16%		
... No qualifications	0	0%		2	0%		
t0_leftright	152	5	2.4	577	4.8	2.4	F=0.47
t0_closeness_labour	158	4.2	3.3	590	4.3	3.4	F=0.236
t0_closeness_conservative	158	4.2	3.5	591	4.1	3.4	F=0.215
t0_policy_proimmigration	146	2.6	1.3	572	2.7	1.3	F=0.255
t0_policy_proredistribution	151	3.2	1.3	575	3.3	1.3	F=0.008
Statistical significance markers: * p<0.1; ** p<0.05; *** p<0.01							

Supplementary Figure 2: Balance table: likelihood of being in the sample by treatment assignment and covariates

B.2 Attrition

12 participants waited a few minutes for a match and then returned their submission without telling me why. It is most plausible that these participants did not find a match, or encountered a bug, given how often this happened to other participants. Another 15 respondents were matched, sometimes sent a few messages, but then left the discussion without contacting me. I checked all the discussions and all but two seem normal. I take an expansive operationalization of attrition and assume that all 28 respondents attritted. Supplementary Fig. 3 shows that the likelihood of attrition cannot be predicted by treatment assignment or pre-treatment covariates, though note that twice as many people attritted when assigned to the treatment condition (18 versus 9).

Include_in_analysis?	0			1			
Variable	N	Mean	SD	N	Mean	SD	Test
Treatment	595			27			X2=2.587
... Control	304	51%		9	33%		
... Treatment	291	49%		18	67%		
t0_gender	591			27			X2=1.299
... Female	339	57%		19	70%		
... Male	252	43%		8	30%		
education	591			27			X2=2.263
... Higher secondary	171	29%		8	30%		
... Bachelor's degree	239	40%		12	44%		
... Lower	38	6%		2	7%		
... Lower secondary	23	4%		2	7%		
... Higher national certificate, NVQ level 4	23	4%		1	4%		
... Master/PhD	95	16%		2	7%		
... No qualifications	2	0%		0	0%		
t0_leftright	577	4.8	2.4	25	4.8	2.4	F=0.001
t0_closeness_labour	590	4.3	3.4	27	5.1	3.4	F=1.187
t0_closeness_conservative	591	4.1	3.4	27	3.3	3	F=1.476
t0_policy_proimmigration	572	2.7	1.3	25	3	1.4	F=0.921
t0_policy_proredistribution	575	3.3	1.3	27	3.2	1.1	F=0.071
Statistical significance markers: * p<0.1; ** p<0.05; *** p<0.01							

Supplementary Figure 3: Balance table: liklihood of attrition by treatment assignment and covariates

B.3 Balance table, manipulation check

The balance table 2 shows that participants assigned to treatment and control are the same on baseline covariates. The manipulation worked as intended. On the platform, all participants selected the party they said they intended to vote for in the pre-screener. All participants sent at least one message, with a mean of 7.3 messages and a standard deviation of 4.2. 96% of participants remembered the party of their discussion partner when asked at the end of the post-treatment survey. 62% correctly remembered their discussion partner's opinion on the statement, and 83% either remembered correctly, or was only off by one point on the five-point Likert scale.

Supplementary Table 2: Probability of treatment assignment by pre-treatment variables

	Probability of assignment to treatment
Intercept	1.511^{***}
	(0.111)
Male	0.046
	(0.043)
Bachelors degree	−0.008
	(0.052)
Higher education	−0.096
	(0.092)
Fewer than 5 GCSEs	−0.031
	(0.114)
Higher national certificate	0.101
	(0.112)
Master's degree/PhD	−0.032
	(0.065)
No qualifications	−0.004
	(0.360)
Closeness Labour	−0.003
	(0.010)
Closeness Conservative	−0.006
	(0.010)
Immigration	0.008
	(0.019)
Redistribution	−0.004
	(0.019)
R^2	0.008
Adj. R^2	−0.011
Num. obs.	582

$^{***}p < 0.001$; $^{**}p < 0.01$; $^{*}p < 0.05$

B.4 All survey questions

Survey 1: Pre-screener survey *** Title 'Brief questionnaire, selection for longer study'; Compensation £0.27, 2 minutes***

[Consent form]

Q1. Please fill in your Prolific ID.

Q2. Are you male or female? Male (1) Female (2)

Q3. What is the highest level of education you have completed? No qualifications, left school at 12 or younger (1) Fewer than 5 good GCSEs, CSEs or O-levels; fewer than 5 good Ordinary or Standard grades (In Scotland); NVQ level 1 (2) 5 or more good GCSEs, CSEs or O-levels, or any A/AS levels; 5 or more good Ordinary or Standard, or any Higher or Advanced Higher grades (In Scotland); NVQ level 2 or 3 (3) Higher national certificate, NVQ level 4 (4) Diploma of Higher Education, Higher National Diploma, NVQ level 5 (5) Bachelor's degree (6) Master's degree, postgraduate diploma or certificate (e.g. PGCE), PhD (7)

Q4. To which of these ethnic groups do you consider you belong? White British (1) Any other white background (2) White and Black Caribbean (3) White and Black African (4) White and Asian (5) Any other mixed background (6) Indian (7) Pakistani (8) Bangladeshi (9) Any other Asian background (10) Black Caribbean (11) Black African (12) Any other black background (13) Chinese (14) Other ethnic group (15) I'd rather not answer (16)

Q5. Thinking back to the General Election in December 2019, do you remember which party you voted for then – or perhaps you didn't vote? Did not vote (1) Conservative Party (2) Labour party (3) Liberal Democrats (4) Scottish National Party (5) Plaid Cymru (6) Green Party (7) Other (8) Brexit Party/Reform UK (9) Don't know (10)

Q6. How strongly or weakly are you attached to this party? Very strongly (1) Strongly (2) Moderately (3) Weakly (4) Very weakly (5) Don't know

Q7. If there were a UK Election tomorrow, which party would you vote for? I would not vote (1) Conservative Party (2) Labour party (3) Liberal Democrats (4) Scottish National Party (5) Plaid Cymru (6) Green Party (7) Other (8) Brexit Party/Reform UK (9) Don't know (10)

Q8. How strongly or weakly are you attached to this party? Very strongly (1) Strongly (2) Moderately (3) Weakly (4) Very weakly (5) Don't know

Q9. How close do you feel to the following political parties (0 – not very close; 10 – very close) Labour Party (1) Conservative Party (2) Liberal Democrats (3) Greens (4)

Q10. In politics, people sometimes talk of left and right. Where would you place yourself on the following scale? (0 – Left; 10 – Right)

Q11. In general terms, how interested in politics are you? Extremely interested (1) Very interested (2) Moderately interested (3) Slightly interested (4) Not interested at all (5) Don't know (6)

Q12. Please tell me how much you agree or disagree with the following statements (Strongly agree (1) – Strongly disagree (5), don't know) (statements randomized) We should change society to deal with issues like gender and racial equality (1) The UK should allow many more immigrants to come to the UK and live (2) Government should reduce inequality

by redistributing income and wealth from the better off to those who are less well off (3) The inheritance tax should be increased (4) Brexit has been bad for the UK (5)

Q13. We would like to select several people to participate in a follow-up study of about 18 minutes. The study will take place in the next few days and you will receive around £ 2 in compensation. For this study, we want to ask you a number of questions again, and ask you to have a brief, anonymous chat conversation on a novel chat platform. Would you be willing to participate? Yes (1) No (2)

Q14. (if 'Yes' to the previous question): Thank you for your willingness to participate. It is very important that you take this study at a pre-agreed time (emphasis in original question). We would therefore like to ask you whether you would be available for the study on the timeslots indicated below. You can fill in multiple timeslots. Timeslots changed with every recruitment wave. There were two or three options, always in one of the next days Timeslot 1 (1) Timeslot 2 (2) Neither (3)

*** Survey 1 END***

Survey 2: Main experiment

Title: Follow-up study [TIMESLOT]; Compensation £2, 18 minutes

[Invitation 1 experiment in private message, sent out the day before] 'Dear participant, You have indicated to be available for an 18-minute study at [TIMESLOT]. I will make the study available on Prolific during the day tomorrow but please, do not start the study earlier or later than [TIMESLOT]. Thank you for your participation! Kind regards, Jona'

[Invitation 2 experiment in private message, sent out 30 minutes before] 'Dear participant, Just a friendly reminder that the study titled '[TITLE]' will start in 30 minutes at [TIMESLOT] sharp. Please be there on time. Thank you! Kind regards, Jona'.

[Description of study to participants before taking the study] We ask you to start this study AT EXACTLY [TIMESLOT]. Please do not start the study earlier or later, you will not be able to participate if you do. The study will ask you to go to a chat platform, have an anonymous chat conversation with another participant and answer a few questions. Though the platform should work on a mobile phone, both the platform and the message writing work best on a laptop/personal computer. Thank you for your participation!

[Consent form]

Q1. Please fill in your Prolific ID below. Note that you will only receive full compensation for your participation if you finish the study.

[Random assignment to treatment or control condition. Participants in the control condition first answers questions 2 – 8. Those in the treatment conditions went straight to question 9]

Q2. (Control). Welcome! We would first like to ask you a few questions

Q3. (Control) Affective polarization. We would like to ask you how you feel about the following groups (0 – 10/strongly dislike – strongly like) (groups randomized) Labour Party voters Conservative Party voters Liberal Democrat voters Green Party voters

Q4. (Control) Political tolerance. We would now like to present you with two statements about the group you like the least. Please first indicate below which of the following groups you like the least. We will then present you with two statements. Labour Party voters (1) Conservative Party voters (2) Liberal Democrat voters (3) Green Party voters (4) Leavers

(5) Remainers (6)

Q5. (Control) Political tolerance. Please indicate how much you agree or disagree with the following statement (0 – 10/Strongly disagree – Strongly agree) (statements randomized) Members of this group should be banned from holding protest in your local area. Members of this group should be allowed to make a speech in your local area.

Q6. (Control) We now have two more general questions. (questions Q7 and Q8 randomized)

Q7. (Control) How comfortable would you be with having a discussion about politics with someone from the other side of the political spectrum (0 – 10/completely uncomfortable – completely comfortable)?

Q8. (Control) How comfortable would you be with having close friends from the other side of the political spectrum (0 – 10/completely uncomfortable – completely comfortable)?

Q9. 'Thank you for filling in the questions! (control)/Welcome! (treatment).

In a few moments, you will be matched to someone from a different political party in order to have an anonymous chat conversation. We will now explain how this works, so please read this text carefully. In the next screen, you will be able to select the political party that you support. We would like you to pick the party that you would vote for were elections to be held today. After you have made your choice, you will be matched to someone from another political party.

The matching process can take a few minutes, please do not close or refresh your browser while you wait. When you have been matched, we will provide you with a statement. The conversation will be about this statement. After you have given your opinion about the statement, the conversation will start. The conversation will last for 10 minutes. We want to ask you to keep conversing for the entire 10 minutes. That means not doing something else while conversing, and responding to your conversation partner. In the chatroom, on the right, there is a clock that counts down from 10 minutes. When the clock reaches 00.00, you will automatically receive a message indicating that the conversation is over. The message reads 'finish, to results'.

!!! Please make sure to click on this message reading ' finish, to results' !!!

Clicking on ' finish, to results' takes you to a series of questions we would like to ask you after the conversation. We would again like to ask you to not close or refresh your browser. Is everything clear so far? Yes (1) No (2)

TREATMENT

Matching screen info text (delivered to participants while waiting for a match) You will now be matched to someone who supports another political party. This can take a few minutes, please do not close or refresh your browser. If it takes more than 5 minutes, please go back to Prolific and let me know.

When you have been matched, you will automatically be led to our chat room. First, you will be presented with a statement and asked to give your opinion. In the chatroom, on the right, you will be able to see the party your discussion partner supports, and his/her opinion on the statement. Also on the right, there is a timer that counts down from 10 minutes. We expect you to keep the conversation going for the whole 10 minutes. When the timer reaches 00.00, the conversation is over.

!!! When the conversation is over, you will be asked to click on a button that reads ’finish, to results’ and answer a few more questions to receive your compensation !!!

Q10. Please indicate how much you agree or disagree with the following statement. After, the conversation will start (1-5/strongly agree – Strongly disagree). One of the following two statements, randomly assigned. Government should reduce inequality by redistributing income and wealth from the better off to those who are less well off. The UK should allow many more immigrants to come to the UK and live.

Chatroom intro text (delivered while in the chatroom) Welcome to the chatroom. Please have a discussion about the statement provided. When the timer hits 00.00 - click on the message that reads ’finish, to results’ to answer a few final questions

After conversation text (delivered after the conversation) The conversation is over. Please click on ’ finish, to results’ to answer a few questions and receive your compensation

POST-CONVERSATION SURVEY

Q11. The conversation is over. We would like to ask you a couple of questions. This will take a few minutes. After, you can close your browser.

[Exact repetition of questions 2-8]

Q12. We would now like to again ask you for your opinion on two statements. How much do you agree or disagree with the following statements (1-5/Strongly agree-strongly disagree) The UK should allow many more immigrants to come to the UK and live. Government should reduce inequality by redistributing income and wealth from the better of to the worse off

Q13. Finally, we would like to ask you about the conversation itself. We would first like to ask you to write down how you experienced the conversation. Try to be as specific as possible, for instance by giving examples. Also make sure to mention both positive and negative aspects of the conversation, if those apply. Write down at least five sentences in the field below. The more details the better!

Q14. Now, we have a couple more statements about the conversation. Please indicate how much you agree or disagree with these statements, where 0 indicates ‘strongly disagree’ and 10 indicates ‘strongly agree’. (statements below presented in random order). I enjoyed the conversation My discussion partner did not have good arguments for his/her opinion The discussion made me see things from the perspective of my discussion partner My discussion partner wanted to hear what I had to say My discussion partner and I don’t have a lot in common My discussion partner was primarily interested in winning the argument My discussion partner did not engage with my arguments

Q14. What party did your discussion partner support? (manipulation check) Labour Party (1) Conservative Party (2) Liberal Democrats (3) Green Party (4) I don’t remember (5)

Q15. What was your discussion partner’s opinion on the statement you discussed? (manipulation check) Strongly agree (1) Agree (2) Neither agree nor disagree (3) Disagree (4) Strongly disagree (5) I don’t remember (6)

Q16. In about two weeks, we would like to again ask you a couple of questions. Answering will take a few minutes and you will be paid £0.50. Would you be willing to be contacted for this study using your Prolific ID on the Prolific website? Yes please (1) No thank you

(2)

Q17. At some point in the future, we would like to make this platform freely available to the public. If that happens, would you be willing to use it without getting paid? Yes (1) No (2)

Q18. And would you recommend it to your friends and family? Yes (1) No (2)

Q19. The next screen will be the final screen. There, you find some details about the conversation should you be interested. After, the study is over and you can close your browser. If you would like to receive more information about this study, please click on this link to a google document. In a few weeks, the document will contain more information about the study

Thank you for having participated in the study. The purpose of the study was to investigate the effect of a brief, anonymous chat conversation between political opposites on political polarization. To that end, you were matched with a political opposite. If you indicated to vote for the Labour Party, an algorithm matched you with someone from the Conservative Party, and vice versa. If you have any further questions, do not hesitate to contact me using the Prolific messenger service, or at jona.dejong@eui.eu.

Survey 2 End

Survey 3: Durability check

Title: brief survey on politics and society; Compensation £0.67, 5 minutes

This survey was made available on Prolific to participants who completed the second survey. It was sent out between two and three weeks after the second study.

[Consent form]

[Exact repetition of questions 2-8 in survey 2]

Q2. Please tell me how much you agree or disagree with the following statements (Strongly agree (1) – Strongly disagree (5), don't know) (statements randomized) We should change society to deal with issues like gender and racial equality (1) The UK should allow many more immigrants to come to the UK and live (2) Government should reduce inequality by redistributing income and wealth from the better off to those who are less well off (3) The inheritance tax should be increased (4) Brexit has been bad for the UK (5)

Q3. On a scale from one to ten, where 'one' means never and 'ten' means all the time, how often do you talk to the following groups of people face-to-face or over the phone about political issues or candidates?" Labour Party voters (1) Conservative Party voters (2)

Q4. A few weeks ago, you participated in a study that asked you to have a conversation about a political subject with someone from another party (either a Labour supporter or a Conservative supporter). Do you remember participating in this study? Yes (1) No (2)

Q5. If 'Yes' under Q4: Have you thought about this conversation at all since you participated? Yes (1) No (2)

Q6. If 'Yes' under Q5: Please describe in as much detail as you can any thoughts you had about this conversation in the last weeks. What were they about? Please try to give examples and make sure to mention both positive and negative aspects, if that applies.

Q7. For all: When you think about the conversation now, how do you remember it? Please describe in detail below and try to give examples and mention both positive and negative aspects.

C Supplementary analysis

C.1 Supplementary Methods 1. Affective polarization (Fig. 3 main paper)

C.1.1 Regression table Fig. 3

Table 3 shows average treatment effects and persistence of treatment effects of the discussion.

Supplementary Table 3: Figure 3 main paper

	Sympathy	Discussion	Friends	Sympathy 2-3 weeks	Discussion 2-3 weeks	Friends 2-3 weeks
(Intercept)	4.253***	5.191***	9.316***	3.800***	5.064***	9.010***
	(0.599)	(0.724)	(0.717)	(0.553)	(0.742)	(0.717)
TreatmentTreatment	1.192***	1.188***	0.496*	0.186	0.531*	−0.118
	(0.178)	(0.199)	(0.204)	(0.156)	(0.208)	(0.210)
t0_education	−0.001	0.009	−0.150*	−0.024	−0.031	−0.217**
	(0.056)	(0.063)	(0.066)	(0.050)	(0.068)	(0.066)
t0_ethnicity	−0.124	−0.232	0.394	0.490	0.143	0.795*
	(0.374)	(0.368)	(0.356)	(0.345)	(0.402)	(0.374)
t0_politicalinterest	−0.099	0.710***	0.032	−0.071	0.718***	0.037
	(0.094)	(0.117)	(0.133)	(0.093)	(0.121)	(0.135)
t0_outparty_closeness	0.442***	0.030	0.027	0.460***	0.007	0.005
	(0.052)	(0.051)	(0.061)	(0.055)	(0.056)	(0.061)
partystrength	−0.490***	−0.306*	−0.698***	−0.548***	−0.314*	−0.618***
	(0.112)	(0.118)	(0.149)	(0.115)	(0.138)	(0.155)
t0_genderMale	−0.166	0.573**	0.618**	−0.048	0.503*	0.620*
	(0.182)	(0.213)	(0.228)	(0.158)	(0.232)	(0.239)
R^2	0.278	0.159	0.085	0.294	0.107	0.078
Adj. R^2	0.269	0.149	0.074	0.285	0.096	0.066
Num. obs.	575	574	574	552	550	550
RMSE	2.003	2.396	2.504	1.798	2.505	2.532
N Clusters	297	297	297	296	296	296

$^{***}p < 0.001$; $^{**}p < 0.01$; $^{*}p < 0.05$

C.1.2 Within-individual effects of the discussion on sympathy, friends and discussion

Treatment effects in the between-design hold with a different identification strategy. I use a within-design, using only participants randomly assigned to the control condition, who filled in the outcome measures before and after the discussion. Paired-sample t tests compare their answers to the outcome measures before and after the discussion. Another set of t tests compare their answers before the discussion and two-three weeks later, to see whether effects persisted. Supplementary Fig.4 shows the results. They are largely similar to the results in Fig. 3 in the main paper. (Point estimates show the mean difference between the groups, bars are 95% Confidence Intervals).

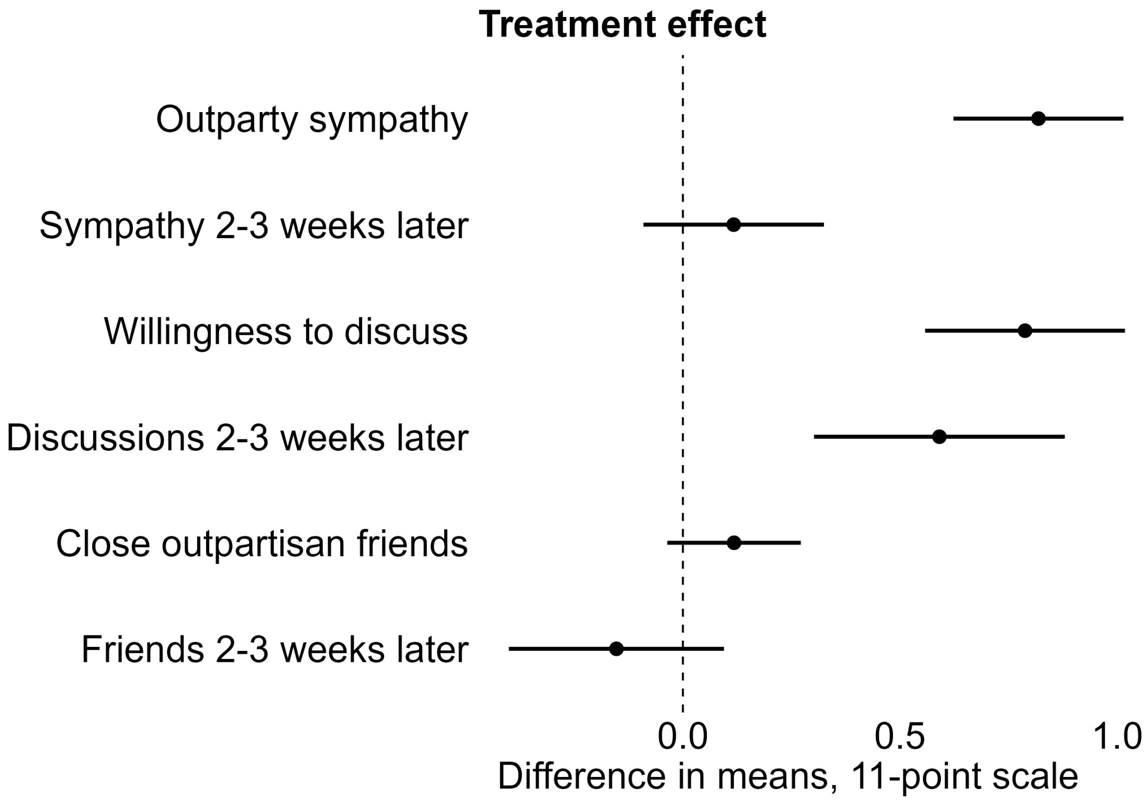

Supplementary Figure 4: Within-effects of the discussion on affective polarization. Error bars are 95% CIs.

C.1.3 Placebo test

There are two alternative explanations for observed treatment effects. First, it is possible that participants assigned to the waitlist control group give relatively low scores because they are anticipating a chat discussion. In that case, the observed effect of the discussion actually shows differences between a control group impacted by anticipation, and a treatment group not impacted by the discussion experience. Second, perhaps the discussion itself does not affect outcomes. Instead, they are the result of other facets of the experience, like generally participating in a research study, having to think about out-partisans, or demand effects.

To further engage with the potential anticipation effect, I conduct a placebo test below. Participants were not only asked about affect towards Labour and Conservative voters, with whom they would eventually have a discussion. They were also asked about affect towards Green party voters, and Liberal Democrat party voters, in order to lower suspicions about the purpose of the study. If there is a general anticipation effect, those in the control condition should also give a more negative rating to inparty, Green and Lib-dem voters, and there should be an observable treatment effect of the discussion on in-party, Green and Lib-dem sympathy. To see whether there is, I run the same between-model used to make Fig. 3 in the main paper. Of course, a positive effect in these models could also show a spill-over effect of the treatment. Perhaps a discussion with a Labour participant also effects one's sympathy towards Green party voters. The Supplementary figure below shows the effect of the treatment on in-party, Green and Lib-dem sympathy.

Supplementary Fig. 5 shows that there is no general affect of the treatment on either outcome. Further breaking this down by party shows a small increase in sympathy towards Green voters among respondents who vote Conservative. If there was a general anticipation effect, most if not all results should be significant. The effect on Green voters is interesting and should serve as an encouragement to study spill-overs towards other parties of depolarization interventions in multiparty systems. The only effects that are found are far smaller than the observed effect on out-party sympathy. (Point estimates show the mean difference between the groups, bars are 95% Confidence Intervals)

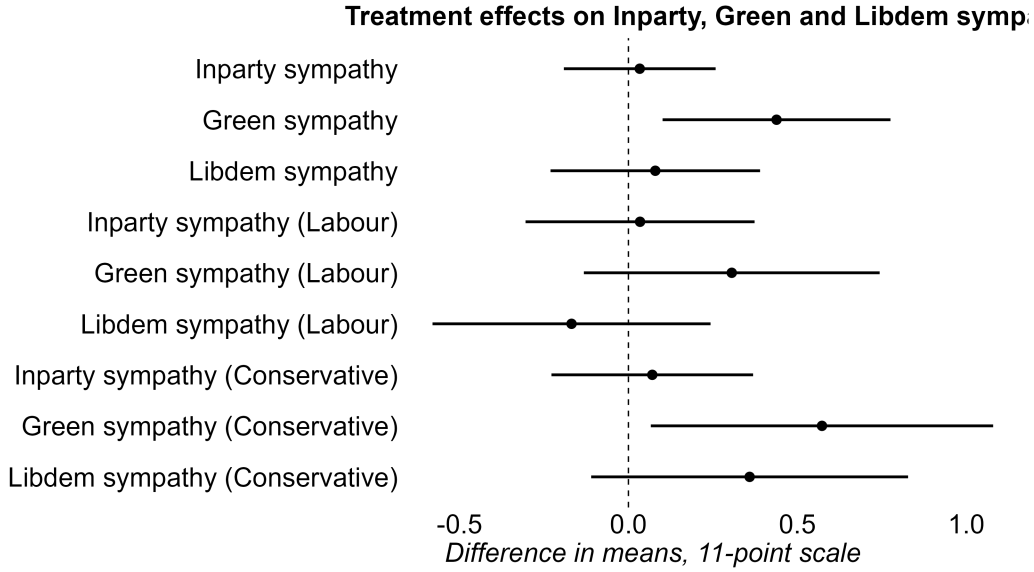

Supplementary Figure 5: Placebo test: discussion effects on inparty, green and lib-dem sympathy. Error bars are 95% CIs.

C.1.4 Results by party

Supplementary Table 4: Treatment effects by party

	Sympathy	Discussion	Friends
(Intercept)	4.531***	5.284***	9.822***
	(0.602)	(0.754)	(0.708)
TreatmentTreatment	1.092***	1.165***	0.314
	(0.222)	(0.292)	(0.244)
PartyLabour	−0.913***	−0.317	−1.692***
	(0.208)	(0.310)	(0.292)
t0_education	0.031	0.021	−0.091
	(0.056)	(0.062)	(0.064)
t0_ethnicity	−0.338	−0.310	−0.006
	(0.373)	(0.375)	(0.361)
t0_politicalinterest	−0.079	0.717***	0.071
	(0.091)	(0.117)	(0.126)
t0_genderMale	−0.178	0.567**	0.591**
	(0.176)	(0.213)	(0.217)
t0_outparty_closeness	0.427***	0.025	0.000
	(0.051)	(0.051)	(0.057)
partystrength	−0.435***	−0.286*	−0.596***
	(0.112)	(0.121)	(0.146)
TreatmentTreatment:PartyLabour	0.214	0.051	0.387
	(0.305)	(0.409)	(0.400)
R^2	0.306	0.162	0.164
Adj. R^2	0.295	0.149	0.151
Num. obs.	575	574	574
RMSE	1.967	2.396	2.397
N Clusters	297	297	297

$^{***}p < 0.001$; $^{**}p < 0.01$; $^{*}p < 0.05$

C.2 Supplementary Methods 2. Discussion effects by issue disagreement (Fig. 4 main paper)

C.2.1 Randomization check

Table 5 shows that issue distance is uncorrelated with treatment assignment and pre-treatment covariates. Just like in fig. 4 in the main paper, I look at respondents with strong opinions and moderate opinions separately.

Supplementary Table 5: Disagreement by treatment status and pre-treatment covariates

	Strong opinion	Moderate opinion
(Intercept)	1.923**	1.208**
	(0.634)	(0.379)
TreatmentTreatment	0.183	−0.174
	(0.204)	(0.121)
t0_education	0.040	−0.000
	(0.062)	(0.039)
t0_ethnicity	0.084	0.363
	(0.413)	(0.235)
t0_politicalinterestModerately interested	0.189	0.096
	(0.302)	(0.210)
t0_politicalinterestNot interested at all	−0.402	−0.159
	(0.606)	(0.337)
t0_politicalinterestSlightly interested	0.127	−0.095
	(0.412)	(0.244)
t0_politicalinterestVery interested	−0.030	0.157
	(0.288)	(0.212)
t0_genderMale	−0.037	0.028
	(0.213)	(0.130)
R^2	0.018	0.028
Adj. R^2	−0.029	−0.002
Num. obs.	177	273

$^{***}p < 0.001$; $^{**}p < 0.01$; $^{*}p < 0.05$

C.2.2 Regression tables and interaction models figure 4

An equivalence test was conducted with the R package 'TOSTER', to test whether the observed effect of the treatment for discussants with a distance of 3 or 4 positions is at least as extreme as the smallest effect size of interest (SESOI). Determining this effect size is somewhat arbitrary. In my case, I take the effect size (Cohen's d.) for discussants with a distance of 0, 1, or 2 positions as the SESOI, which is 0.65. This yields a P-value of 0.06.

Supplementary Table 6: Figure 4 - Outcomes by issue distance

	Sympathy	Discussion	Friends
(Intercept)	3.587^{***}	5.208^{***}	8.812^{***}
	(0.721)	(0.895)	(0.897)
TreatmentTreatment	1.732^{***}	1.313^{**}	0.457
	(0.344)	(0.448)	(0.386)
Distance_cont_t0	0.001	0.118	0.062
	(0.118)	(0.176)	(0.149)
t0_education	0.007	−0.012	-0.143^{*}
	(0.058)	(0.066)	(0.068)
t0_ethnicity	−0.034	−0.309	0.351
	(0.370)	(0.371)	(0.380)
t0_politicalinterest	−0.065	0.688^{***}	0.023
	(0.096)	(0.122)	(0.137)
t0_genderMale	−0.179	0.621^{**}	0.610^{*}
	(0.188)	(0.221)	(0.237)
opinion_t0	0.170	−0.023	0.225^{*}
	(0.090)	(0.103)	(0.107)
t0_outparty_closeness	0.406^{***}	0.015	−0.012
	(0.052)	(0.052)	(0.066)
partystrength	-0.455^{***}	-0.264^{*}	-0.701^{***}
	(0.123)	(0.125)	(0.165)
TreatmentTreatment:Distance_cont_t0	-0.320^{*}	−0.135	−0.013
	(0.158)	(0.206)	(0.207)
R^2	0.298	0.155	0.092
Adj. R^2	0.284	0.139	0.075
Num. obs.	537	536	536
RMSE	2.001	2.388	2.508
N Clusters	270	270	270

$^{***}p < 0.001$; $^{**}p < 0.01$; $^{*}p < 0.05$

Supplementary Table 7: Figure 4 - Sympathy by distance and intial opinion

	Strong opinion	Moderate opinion	Neutral
(Intercept)	3.005**	4.122***	5.472*
	(1.071)	(0.994)	(2.195)
TreatmentTreatment	2.245**	1.698**	0.882
	(0.647)	(0.496)	(1.373)
Distance_cont_t0	0.084	−0.074	−0.385
	(0.144)	(0.179)	(0.736)
t0_education	0.042	−0.030	0.055
	(0.096)	(0.084)	(0.137)
t0_ethnicity	0.701	−0.233	−0.396
	(0.587)	(0.567)	(1.081)
t0_politicalinterest	−0.138	−0.006	−0.045
	(0.174)	(0.138)	(0.343)
t0_genderMale	−0.287	0.012	−0.063
	(0.318)	(0.262)	(0.543)
t0_outparty_closeness	0.550***	0.399***	0.235
	(0.082)	(0.077)	(0.139)
partystrength	−0.496*	−0.422*	−0.539
	(0.227)	(0.162)	(0.328)
TreatmentTreatment:Distance_cont_t0	−0.502*	−0.327	0.171
	(0.242)	(0.253)	(0.876)
R^2	0.388	0.260	0.183
Adj. R^2	0.355	0.235	0.090
Num. obs.	175	273	89
RMSE	1.921	2.029	2.176
N Clusters	146	204	84

$^{***}p < 0.001$; $^{**}p < 0.01$; $^{*}p < 0.05$

The following tables show the results of Fig. 4 with a dummy version of the baseline issue distance variable, that take the value (1) if one respondent (strongly) disagrees and the other (strongly) agrees, and 0 otherwise.

Supplementary Table 8: Figure 4 - Outcomes by issue distance (dummy variable)

	Sympathy	Discussion	Friends
(Intercept)	3.478***	5.207***	8.872***
	(0.677)	(0.815)	(0.838)
TreatmentTreatment	1.503***	1.217***	0.353
	(0.244)	(0.300)	(0.279)
Distance_t0_dummy	0.099	0.403	0.097
	(0.229)	(0.319)	(0.293)
t0_education	0.006	−0.011	−0.141*
	(0.058)	(0.065)	(0.067)
t0_ethnicity	−0.062	−0.320	0.345
	(0.370)	(0.370)	(0.382)
t0_politicalinterest	−0.069	0.681***	0.023
	(0.096)	(0.121)	(0.137)
t0_genderMale	−0.190	0.616**	0.601*
	(0.189)	(0.219)	(0.235)
opinion_t0	0.204*	−0.002	0.237*
	(0.086)	(0.098)	(0.110)
t0_outparty_closeness	0.409***	0.014	−0.014
	(0.053)	(0.053)	(0.066)
partystrength	−0.444***	−0.258*	−0.707***
	(0.123)	(0.126)	(0.163)
TreatmentTreatment:Distance_t0_dummy	−0.753*	−0.313	0.194
	(0.360)	(0.409)	(0.425)
R^2	0.295	0.157	0.093
Adj. R^2	0.282	0.141	0.076
Num. obs.	537	536	536
RMSE	2.005	2.385	2.506
N Clusters	270	270	270

$^{***}p < 0.001$; $^{**}p < 0.01$; $^{*}p < 0.05$

Supplementary Table 9: Figure 4 - Sympathy by distance (dummy variable) and intial opinion

	Strong opinion	Moderate opinion
(Intercept)	2.982^{**}	4.127^{***}
	(1.062)	(0.955)
TreatmentTreatment	1.932^{***}	1.453^{***}
	(0.422)	(0.387)
Distance_t0_dummy	0.632	−0.248
	(0.388)	(0.331)
t0_education	0.031	−0.025
	(0.096)	(0.086)
t0_ethnicity	0.614	−0.259
	(0.610)	(0.571)
t0_politicalinterest	−0.169	−0.012
	(0.175)	(0.136)
t0_genderMale	−0.268	0.005
	(0.319)	(0.265)
t0_outparty_closeness	0.549^{***}	0.403^{***}
	(0.084)	(0.078)
partystrength	−0.454	-0.413^{*}
	(0.232)	(0.162)
TreatmentTreatment:Distance_t0_dummy	-1.679^{**}	−0.490
	(0.609)	(0.508)
R^2	0.391	0.257
Adj. R^2	0.358	0.232
Num. obs.	175	273
RMSE	1.916	2.033
N Clusters	146	204

$^{***}p < 0.001$; $^{**}p < 0.01$; $^{*}p < 0.05$

This table shows a three-way interaction between the treatment, baseline opinion strength (strong or moderate) and disagreement (using the dummy version of the variable).

Supplementary Table 10: Figure 4 - Sympathy by distance (dummy variable) * intial opinion

	Three-way interaction model figure 4
(Intercept)	4.014^{***}
	(0.735)
TreatmentTreatment	1.421^{***}
	(0.389)
Distance_t0_dummy	−0.259
	(0.326)
Strong opinion	−0.656
	(0.362)
t0_education	−0.009
	(0.064)
t0_ethnicity	0.050
	(0.427)
t0_politicalinterest	−0.071
	(0.103)
t0_genderMale	−0.122
	(0.203)
t0_outparty_closeness	0.462^{***}
	(0.058)
partystrength	-0.442^{**}
	(0.136)
Treatment:Distance	−0.434
	(0.507)
Treatment:Strong opinion	0.509
	(0.546)
Distance:Strong opinion	0.878
	(0.497)
Treatment:Distance:Strong opinion	−1.246
	(0.750)
R^2	0.315
Adj. R^2	0.294
Num. obs.	448
RMSE	1.986
N Clusters	265

$^{***}p < 0.001$; $^{**}p < 0.01$; $^{*}p < 0.05$

C.3 Supplementary Methods 3. Opinion change (Fig. 5 main paper)

C.3.1 Baseline opinions, and opinions before the discussion, by treatment assignment

Supplementary Table 11: Opinions by treatment assignment

	Baseline opinions	Opinions pre-discussion
(Intercept)	2.333***	2.449***
	(0.068)	(0.059)
TreatmentTreatment	−0.082	0.022
	(0.097)	(0.084)
R^2	0.001	0.000
Adj. R^2	−0.001	−0.002
Num. obs.	567	577

$^{***}p < 0.001$; $^{**}p < 0.01$; $^{*}p < 0.05$

C.3.2 Models figure 5 main paper

Supplementary Table 12: Figure 5 - pre-screener –> pre-discussion

	Pre-discussion	Pre-discussion (Conservative)	Pre-discussion (Labour)
(Intercept)	2.688***	2.496***	2.929***
	(0.309)	(0.494)	(0.392)
TreatmentTreatment	0.185*	0.373**	−0.024
	(0.087)	(0.131)	(0.120)
t0_education	−0.006	0.011	−0.032
	(0.029)	(0.040)	(0.039)
t0_ethnicity	0.149	0.244	0.054
	(0.184)	(0.366)	(0.199)
t0_politicalinterest	−0.104	−0.100	−0.126
	(0.053)	(0.074)	(0.065)
t0_genderMale	0.045	0.173	−0.091
	(0.095)	(0.143)	(0.128)
t0_outparty_closeness	0.130***	0.128***	0.130***
	(0.027)	(0.034)	(0.029)
partystrength	−0.114	−0.151	−0.056
	(0.061)	(0.086)	(0.080)
R^2	0.100	0.107	0.115
Adj. R^2	0.089	0.085	0.092
Num. obs.	566	288	278
RMSE	1.050		
N Clusters	297		

$^{***}p < 0.001$; $^{**}p < 0.01$; $^{*}p < 0.05$

Supplementary Table 13: Figure 5 - Hiding views by party

	Hide views by party
(Intercept)	2.769^{***}
	(0.298)
TreatmentTreatment	0.335^{*}
	(0.134)
PartyLabour	0.054
	(0.140)
t0_education	0.001
	(0.031)
t0_ethnicity	0.018
	(0.192)
t0_politicalinterest	-0.163^{***}
	(0.047)
t0_genderMale	0.114
	(0.096)
TreatmentTreatment:PartyLabour	-0.381^{*}
	(0.187)
R^2	0.037
Adj. R^2	0.025
Num. obs.	569
RMSE	1.089
N Clusters	297

$^{***}p < 0.001$; $^{**}p < 0.01$; $^{*}p < 0.05$

Supplementary Table 14: Figure 5 - pre-discussion –> post-discussion

	Pre-post discussion	Pre-post discussion (C)	Pre-post discussion (L)
(Intercept)	2.883***	2.440***	3.265***
	(0.303)	(0.492)	(0.340)
TreatmentTreatment	−0.093	−0.077	−0.125
	(0.078)	(0.130)	(0.106)
t0_education	−0.036	0.008	−0.081*
	(0.029)	(0.040)	(0.034)
t0_ethnicity	0.192	0.302	0.048
	(0.180)	(0.364)	(0.177)
t0_politicalinterest	−0.090	−0.035	−0.149**
	(0.048)	(0.073)	(0.057)
t0_genderMale	−0.029	0.026	−0.110
	(0.085)	(0.143)	(0.112)
t0_outparty_closeness	0.135***	0.154***	0.107***
	(0.027)	(0.034)	(0.026)
partystrength	−0.110	−0.121	−0.051
	(0.058)	(0.085)	(0.068)
R^2	0.113	0.094	0.149
Adj. R^2	0.102	0.071	0.127
Num. obs.	570	287	283
RMSE	0.997		
N Clusters	297		

$^{***}p < 0.001$; $^{**}p < 0.01$; $^{*}p < 0.05$

Supplementary Table 15: Figure 5 - pre-screener –> post-discussion

	Opinion change discussion	Opinion change (C)	Opinion change (L)
(Intercept)	2.772^{***}	2.491^{***}	3.196^{***}
	(0.315)	(0.500)	(0.400)
TreatmentTreatment	0.034	0.191	−0.146
	(0.085)	(0.132)	(0.123)
t0_education	0.007	0.037	−0.044
	(0.030)	(0.041)	(0.040)
t0_ethnicity	0.167	0.259	0.073
	(0.196)	(0.371)	(0.204)
t0_politicalinterest	-0.153^{**}	−0.140	-0.190^{**}
	(0.053)	(0.075)	(0.067)
t0_genderMale	0.088	0.268	−0.103
	(0.094)	(0.144)	(0.131)
t0_outparty_closeness	0.146^{***}	0.155^{***}	0.134^{***}
	(0.028)	(0.034)	(0.030)
partystrength	-0.129^{*}	-0.182^{*}	−0.057
	(0.062)	(0.087)	(0.082)
R^2	0.123	0.132	0.148
Adj. R^2	0.112	0.110	0.126
Num. obs.	567	289	278
RMSE	1.071		
N Clusters	297		

$^{***}p < 0.001$; $^{**}p < 0.01$; $^{*}p < 0.05$

C.3.3 Conservatives hide their views when about to discuss immigration

Supplementary Table 16: Updating views pre-screener –> pre-discussion by conversation topic for Conservatives

	Hiding views by party and topic
(Intercept)	2.363***
	(0.301)
TreatmentTreatment	0.549**
	(0.165)
PartyLabour	0.897***
	(0.180)
convo_topicRedistribution	0.651***
	(0.169)
t0_education	−0.014
	(0.028)
t0_ethnicity	0.066
	(0.182)
t0_politicalinterest	−0.098*
	(0.049)
t0_genderMale	0.002
	(0.089)
t0_outparty_closeness	0.127***
	(0.027)
partystrength	−0.110
	(0.059)
TreatmentTreatment:PartyLabour	−0.800***
	(0.236)
TreatmentTreatment:convo_topicRedistribution	−0.247
	(0.251)
PartyLabour:convo_topicRedistribution	−1.354***
	(0.245)
TreatmentTreatment:PartyLabour:convo_topicRedistribution	0.632
	(0.337)
R^2	0.169
Adj. R^2	0.150
Num. obs.	566
RMSE	1.014
N Clusters	297

$^{***}p < 0.001$; $^{**}p < 0.01$; $^{*}p < 0.05$

An equivalence test was conducted with the R package 'TOSTER', to test whether the observed effect of the treatment for Labour discussants is at least as extreme as the smallest effect size of interest (SESOI). To determine the SESOI, I take the effect size (Cohen's d.) for Conservative discussants, which is 0.3. This yields a P-value of 0.02.

I conduct a second equivalence test to see whether the observed effect of the treatment for Conservative discussants who discuss Redistribution is at least as extreme as the smallest effect size of interest. This yields a P-value of 0.08.

C.3.4 Robustness figure 5: within-results

Supplementary Table 17: within-results figure 5 main paper

	Opinion_change	Party	Estimate	t.statistic	p.value
1	t0 –> t1.1	Both parties	0.17	-2.59	0.0096
2	t0 –> t1.1	Conserative	0.29	-3.12	0.0019
3	t0 –> t1.1	Labour	0.03	-0.46	0.65
4	t1.1 –> t1.2	Both parties	-0.11	1.67	0.095
5	t1.1 –> t1.2	Conserative	-0.1	1.09	0.28
6	t1.1 –> t1.2	Labour	-0.1	1.34	0.18
7	t0 –> t1.2	Both parties	0.06	-0.91	0.36
8	t0 –> t1.2	Conserative	0.19	-1.92	0.055
9	t0 –> t1.2	Labour	0.07	0.74	0.46

C.3.5 Robustness: view change between pre-screener and follow-up survey

Supplementary Table 18: Placebo: Immigration

	Immigration	Labour	Conservative
(Intercept)	2.702***	3.439***	2.294***
	(0.276)	(0.386)	(0.396)
TreatmentTreatment	0.062	−0.074	0.147
	(0.089)	(0.131)	(0.116)
t0_education	0.016	−0.066	0.033
	(0.029)	(0.044)	(0.034)
t0_ethnicity	−0.038	0.112	0.254
	(0.168)	(0.199)	(0.274)
t0_politicalinterest	−0.046	−0.122	−0.015
	(0.053)	(0.077)	(0.062)
t0_genderMale	−0.086	0.014	−0.190
	(0.100)	(0.145)	(0.124)
t0_outparty_closeness	0.076**	0.088*	0.079*
	(0.029)	(0.037)	(0.033)
partystrength	−0.145*	−0.098	−0.266**
	(0.058)	(0.084)	(0.078)
R^2	0.049	0.082	0.107
Adj. R^2	0.037	0.058	0.084
Num. obs.	553	270	283
RMSE	1.071	1.071	0.959
N Clusters	296	270	283

$^{***}p < 0.001$; $^{**}p < 0.01$; $^{*}p < 0.05$

Supplementary Table 19: Placebo: redistribution

	Redistribution	Labour	Conservative
(Intercept)	2.904***	2.460***	3.369***
	(0.313)	(0.367)	(0.458)
TreatmentTreatment	−0.006	−0.138	0.138
	(0.091)	(0.117)	(0.141)
t0_education	−0.031	−0.036	−0.006
	(0.031)	(0.040)	(0.043)
t0_ethnicity	0.098	0.008	−0.233
	(0.169)	(0.194)	(0.266)
t0_politicalinterest	−0.116*	−0.088	−0.146
	(0.051)	(0.071)	(0.082)
t0_genderMale	0.063	−0.008	0.149
	(0.098)	(0.116)	(0.151)
t0_outparty_closeness	0.148***	0.131**	0.151***
	(0.026)	(0.037)	(0.035)
partystrength	−0.149*	−0.040	−0.173
	(0.058)	(0.081)	(0.088)
R^2	0.120	0.110	0.111
Adj. R^2	0.109	0.086	0.089
Num. obs.	555	274	281
RMSE	1.081	0.959	1.150
N Clusters	297	274	281

$^{***}p < 0.001$; $^{**}p < 0.01$; $^{*}p < 0.05$

Supplementary Table 20: Placebo: tax

	Tax	Labour	Conservative
(Intercept)	3.718^{***}	4.974^{***}	2.788^{***}
	(0.426)	(0.498)	(0.521)
TreatmentTreatment	−0.042	−0.281	0.113
	(0.106)	(0.155)	(0.145)
t0_education	−0.037	−0.057	-0.094^{*}
	(0.038)	(0.051)	(0.045)
t0_ethnicity	-0.693^{*}	−0.559	0.128
	(0.268)	(0.279)	(0.403)
t0_politicalinterest	−0.064	−0.140	−0.045
	(0.072)	(0.089)	(0.086)
t0_genderMale	0.183	0.150	0.183
	(0.124)	(0.172)	(0.160)
t0_outparty_closeness	0.098^{**}	0.119^{**}	0.114^{*}
	(0.030)	(0.035)	(0.043)
partystrength	−0.085	−0.193	−0.164
	(0.079)	(0.099)	(0.097)
R^2	0.061	0.140	0.079
Adj. R^2	0.049	0.116	0.055
Num. obs.	531	254	277
RMSE	1.311	1.248	1.145
N Clusters	295	254	277

$^{***}p < 0.001$; $^{**}p < 0.01$; $^{*}p < 0.05$

Supplementary Table 21: Placebo: SJ

	Social justice	Labour	Conservative
(Intercept)	3.336^{***}	2.147^{***}	3.824^{***}
	(0.306)	(0.287)	(0.513)
TreatmentTreatment	−0.018	0.051	−0.026
	(0.080)	(0.092)	(0.132)
t0_education	−0.046	−0.017	0.014
	(0.031)	(0.032)	(0.041)
t0_ethnicity	0.494^{**}	0.133	0.266
	(0.180)	(0.134)	(0.398)
t0_politicalinterest	-0.155^{**}	−0.097	-0.162^{*}
	(0.053)	(0.055)	(0.073)
t0_genderMale	−0.063	0.250^{*}	-0.395^{**}
	(0.101)	(0.100)	(0.142)
t0_outparty_closeness	0.135^{***}	0.075^{*}	0.158^{***}
	(0.026)	(0.029)	(0.030)
partystrength	-0.240^{***}	−0.084	-0.198^{*}
	(0.064)	(0.056)	(0.092)
R^2	0.148	0.115	0.169
Adj. R^2	0.137	0.092	0.147
Num. obs.	555	275	280
RMSE	1.122	0.752	1.107
N Clusters	297	275	280

$^{***}p < 0.001$; $^{**}p < 0.01$; $^{*}p < 0.05$

Supplementary Table 22: Placebo: Brexit

	Brexit	Labour	Conservative
(Intercept)	2.934***	2.675***	2.763***
	(0.291)	(0.319)	(0.623)
TreatmentTreatment	0.039	0.027	0.079
	(0.094)	(0.103)	(0.146)
t0_education	−0.069*	−0.128**	0.031
	(0.031)	(0.041)	(0.044)
t0_ethnicity	0.553**	0.171	0.552
	(0.202)	(0.154)	(0.538)
t0_politicalinterest	−0.037	−0.050	0.003
	(0.056)	(0.066)	(0.082)
t0_genderMale	−0.230*	−0.088	−0.432**
	(0.106)	(0.110)	(0.164)
t0_outparty_closeness	0.143***	0.084*	0.159***
	(0.027)	(0.033)	(0.040)
partystrength	−0.315***	−0.143	−0.317***
	(0.059)	(0.073)	(0.090)
R^2	0.162	0.158	0.152
Adj. R^2	0.151	0.135	0.130
Num. obs.	533	260	273
RMSE	1.127	0.831	1.205
N Clusters	291	260	273

$^{***}p < 0.001$; $^{**}p < 0.01$; $^{*}p < 0.05$

C.4 Supplementary Methods 4. Mechanisms

C.4.1 Presence of mechanisms not correlated with treatment assignment

Supplementary Table 23: Prevalence of mechanisms by treatment assignment

	Disagreement	pers.-getting	Inclusion	Commonality
(Intercept)	1.775***	5.522***	7.316***	5.821***
	(0.049)	(0.174)	(0.144)	(0.172)
TreatmentTreatment	−0.052	0.151	0.173	−0.041
	(0.069)	(0.247)	(0.205)	(0.245)
R^2	0.001	0.001	0.001	0.000
Adj. R^2	−0.001	−0.001	−0.001	−0.002
Num. obs.	567	573	573	573

$^{***}p < 0.001$; $^{**}p < 0.01$; $^{*}p < 0.05$

C.4.2 Expressed disagreement only predicted by issue disagreement. Levels of disagreement balanced across treatment and control

The first two columns of table 24 show the extent to which expressed disagreement in the discussion is predicted by a range of pre-treatment covariates, including feeling close to Labour/Conservative voting. In both models, only issue distance to one's partner significantly predicts expressed disagreement in the discussion. The first model includes the 'agreement' category in the model and shows that whether respondents end up agreeing, finding common ground or disagreeing is strongly predicted by the issue distance between them. The second model takes out this category, as in both other categories, respondents express disagreement. The results show that whether they end up finding common ground, or solely disagreeing, is only very weakly predicted by issue distance.

The last three columns of the table show that each level of disagreement is balanced by covariates accross treatment and control. The dependent variable is assignment to treatment; the predictor variables are the usual covariates, and the models are subsetted by each stratum (agreement, common ground, disagreement).

Supplementary Table 24: Expressed disagreement, by pre-treatment covariates, and treatment by covariates for each level of disagreement

	With 'agreement'	Without 'agreement	Agreement	CG	Disagreement
(Intercept)	1.383^{***}	2.526^{***}	0.538^{**}	0.257	0.347
	(0.254)	(0.233)	(0.191)	(0.269)	(0.280)
Education	−0.017	-0.061^{**}	−0.005	0.003	−0.012
	(0.022)	(0.020)	(0.019)	(0.028)	(0.027)
Ethnicity	−0.071	0.073	0.038	0.132	0.136
	(0.135)	(0.115)	(0.128)	(0.148)	(0.192)
Mod. interested	0.042	0.072	−0.048	0.051	−0.079
	(0.116)	(0.102)	(0.107)	(0.136)	(0.149)
Not interested	−0.135	−0.031	0.006	0.096	−0.445
	(0.189)	(0.189)	(0.155)	(0.241)	(0.281)
Sligh. interested	0.039	0.087	−0.040	0.033	0.059
	(0.136)	(0.123)	(0.122)	(0.160)	(0.176)
Very interested	0.118	0.136	−0.041	0.051	0.220
	(0.115)	(0.099)	(0.108)	(0.135)	(0.148)
Male	0.057	0.053	−0.002	0.131	0.027
	(0.073)	(0.063)	(0.068)	(0.089)	(0.085)
Closeness Labour	0.008	0.001			
	(0.015)	(0.014)			
Closeness Conse	0.000	−0.010			
	(0.015)	(0.014)			
Distance	0.235^{***}	0.054^{*}			
	(0.030)	(0.027)			
R^2	0.118	0.070	0.002	0.023	0.104
Adj. R^2	0.101	0.034	−0.024	−0.025	0.055
Num. obs.	527	269	277	151	136

$^{***}p < 0.001$; $^{**}p < 0.01$; $^{*}p < 0.05$

C.4.3 Regression models for figure 6 main paper

Table 25, shows the regression models underlying Fig. 6 in the main paper. As in all between-models, I control for pre-treatment covariates and cluster standard errors at the conversation level. The models show that for the sympathy variable, the effect of the treatment depends on the discussion condition.

Supplementary Table 25: Treatment effect, by expressed disagreement

	Sympathy by agreement	Discussion by agreeme
(Intercept)	4.222^{***}	5.113^{***}
	(0.607)	(0.763)
TreatmentTreatment	1.642^{***}	1.436^{***}
	(0.250)	(0.300)
c_agreementCommon ground	−0.096	0.043
	(0.268)	(0.380)
c_agreementDisagreement	−0.241	0.358
	(0.267)	(0.376)
t0_education	−0.031	0.025
	(0.054)	(0.064)
t0_ethnicity	−0.039	−0.332
	(0.379)	(0.368)
t0_politicalinterest	−0.058	0.716^{***}
	(0.094)	(0.119)
t0_genderMale	−0.120	0.569^{*}
	(0.177)	(0.221)
t0_outparty_closeness	0.420^{***}	0.022
	(0.052)	(0.051)
partystrength	-0.461^{***}	-0.319^{**}
	(0.114)	(0.120)
TreatmentTreatment:c_agreementCommon ground	−0.343	−0.211
	(0.411)	(0.485)
TreatmentTreatment:c_agreementDisagreement	-1.679^{***}	−0.708
	(0.408)	(0.500)
R^2	0.328	0.164
Adj. R^2	0.315	0.147
Num. obs.	560	559
RMSE	1.941	2.418
N Clusters	291	291

$^{***}p < 0.001$; $^{**}p < 0.01$; $^{*}p < 0.05$

C.4.4 Regression models for figure 7 main paper

Supplementary Table 26: Sympathy by mechanisms

	Perspective-getting	Inclusion	Commonality	Reduced mispercepti[illegible]
(Intercept)	3.592^{***}	3.535^{***}	3.543^{***}	4.481^{***}
	(0.654)	(0.632)	(0.596)	(0.581)
TreatmentTreatment	0.188	−0.966	−0.143	0.872^{***}
	(0.390)	(0.504)	(0.335)	(0.186)
t1_perspectivetaking	0.083^{*}			
	(0.037)			
t0_education	0.024	0.013	−0.002	−0.017
	(0.056)	(0.054)	(0.052)	(0.054)
t0_ethnicity	0.010	−0.009	−0.002	−0.117
	(0.384)	(0.359)	(0.370)	(0.366)
t0_politicalinterest	−0.099	−0.094	−0.088	−0.113
	(0.092)	(0.091)	(0.088)	(0.094)
t0_genderMale	−0.157	−0.093	−0.128	−0.185
	(0.172)	(0.173)	(0.174)	(0.179)
t0_outparty_closeness	0.414^{***}	0.432^{***}	0.409^{***}	0.455^{***}
	(0.054)	(0.050)	(0.051)	(0.052)
partystrength	-0.490^{***}	-0.481^{***}	-0.470^{***}	-0.510^{***}
	(0.113)	(0.110)	(0.109)	(0.113)
7Treatment:perspectivetaking	0.172^{**}			
	(0.063)			
Inclusioninconversation		0.067		
		(0.038)		
Treatment:inclusion		0.285^{***}		
		(0.065)		
t1_lotsincommon			0.094^{*}	
			(0.037)	
Treatment:common			0.228^{***}	
			(0.053)	
Red. misp.				−0.343
				(0.321)
Treatment:Red. misp				1.885^{***}
				(0.467)
R^2	0.334	0.346	0.366	0.310
Adj. R^2	0.323	0.336	0.355	0.299
Num. obs.	565	565	565	575
RMSE	1.929	1.911	1.883	1.962
N Clusters	296	296	296	297

$^{***}p < 0.001$; $^{**}p < 0.01$; $^{*}p < 0.05$

Supplementary Table 27: Discussion by mechanisms

	Perspective-getting	Inclusion	Commonality	Reduced misperception
(Intercept)	4.602***	4.579***	4.751***	5.336***
	(0.831)	(0.836)	(0.832)	(0.737)
TreatmentTreatment	1.431***	0.385	0.816	0.999***
	(0.424)	(0.643)	(0.436)	(0.212)
t1_perspectivetaking	0.070			
	(0.050)			
t0_education	0.035	0.031	0.025	0.009
	(0.063)	(0.063)	(0.064)	(0.062)
t0_ethnicity	−0.190	−0.193	−0.205	−0.287
	(0.369)	(0.376)	(0.375)	(0.374)
t0_politicalinterest	0.728***	0.736***	0.734***	0.748***
	(0.118)	(0.117)	(0.118)	(0.115)
t0_genderMale	0.580**	0.606**	0.588**	0.492*
	(0.216)	(0.216)	(0.217)	(0.214)
t0_outparty_closeness	0.030	0.033	0.025	0.016
	(0.052)	(0.052)	(0.052)	(0.051)
partystrength	−0.325**	−0.318**	−0.318**	−0.287*
	(0.120)	(0.120)	(0.121)	(0.117)
Treatment:perspectivetaking	−0.042			
	(0.069)			
t1_inclusioninconversation		0.049		
		(0.055)		
Treatment:tinclusion		0.108		
		(0.083)		
t1_lotsincommon			0.045	
			(0.046)	
Treatment:common			0.067	
			(0.067)	
Red. misp.				−1.314**
				(0.424)
Treatment:Red. misp.				1.088*
				(0.537)
R^2	0.168	0.175	0.173	0.178
Adj. R^2	0.154	0.162	0.159	0.165
Num. obs.	565	565	565	574
RMSE	2.396	2.385	2.389	2.374
N Clusters	296	296	296	297

$^{***}p < 0.001$; $^{**}p < 0.01$; $^{*}p < 0.05$

Supplementary Table 28: Treatment effect by pre-treatment closeness to outparty

	Sympathy by closeness
(Intercept)	4.064^{***}
	(0.609)
TreatmentTreatment	1.540^{***}
	(0.220)
t0_outparty_closeness	0.557^{***}
	(0.065)
t0_education	-0.001
	(0.056)
t0_ethnicity	-0.140
	(0.364)
t0_politicalinterest	-0.094
	(0.093)
t0_genderMale	-0.172
	(0.180)
partystrength	-0.488^{***}
	(0.110)
TreatmentTreatment:t0_outparty_closeness	-0.235^{*}
	(0.098)
R^2	0.289
Adj. R^2	0.278
Num. obs.	575
RMSE	1.990
N Clusters	297

$^{***}p < 0.001$; $^{**}p < 0.01$; $^{*}p < 0.05$

C.5 Supplementary Methods 5. Further pre-registered hypotheses and deviations from the pre-analysis plan

C.5.1 The moderating effect of out-party closeness and ideology

Hypothesis 5 of the PAP reads: 'Perceived distance to the party of your discussion partner will either not affect, or increase the depolarizing effect of political discussions'. Table 28 shows the same between model that was used to make Fig. 3 in the main paper, with an interaction term for out-party closeness.

The table shows evidence for H5. The negative interaction term suggests that the treatment effect is weaker for respondents with higher levels of out-party closeness.

Hypothesis 7 reads: 'There may be spill-over effects. A participant who has a discussion with an ideological extremist could view ideological moderates in a more positive light. A participant who has a discussion with an ideological moderate could view ideological extremists in a more negative light'. The hypothesis was meant for a scenario in which I would recruit respondents from multiple parties, and cannot be tested now.

C.5.2 Political tolerance

Hypothesis 2 of the PAP reads: 'Political discussions between political opposites will increase political tolerance'. In addition to the polarization measures, I included a measure of political tolerance. It asks respondents to first name which of a series of groups they like the least: Labour Party voters, Conservative Party voters, Liberal Democrat voters, Green Party voters, Leavers and Remainers. Then, it asks whether members of this group should be (1) banned from holding protests in your local area and (2) allowed to make a speech in your local area. Where this question in its original version also asks about non-political groups, this one only asked about political groups.

Analysis of this measure is complicated by the fact that some respondents filled in a different group before and after the discussion. This outcome in itself does say something about the effect of the treatment on a different measure of affect. Compared to the control group, the treatment group has 4% fewer respondents who report Conservative party voters as their least liked group, 7% fewer respondents who report Labour voters, and 8% more respondents who report to dislike Leave voters. A chi-squared test of independence has a P value of 0.08, providing some evidence that the treatment indeed causes people to switch.

Chi.Square.Statistic	Degrees.of.Freedom	p.value
9.73	5	0.08

C.5.3 Topic

Finally, I look at whether the conversation topic matters for the effect of the discussion on polarization. Table 29 shows that for sympathy, the treatment effect is stronger when participants talk about immigration than when they talk about redistribution. The same effect is not found for the other variables. This seems counter-intuitive, as immigration is generally seen as a more contentious topic. At the same time, the UK still has a relatively high level of economic polarization, which may explain the results. In this sample too, there is more baseline disagreement on redistribution than on immigration. Thus, the result of the topic is somewhat complicated by its conflation with issue distance between discussing partisans. The effect of topic is however interesting and warrants further study.

Supplementary Table 29: Results by conversation topic

	Sympathy	Discussion	Friends
(Intercept)	4.193***	5.073***	9.103***
	(0.590)	(0.771)	(0.741)
TreatmentTreatment	1.596***	1.415***	0.704*
	(0.245)	(0.300)	(0.310)
convo_topicRedistribution	−0.041	0.150	0.357
	(0.215)	(0.309)	(0.266)
t0_education	0.004	0.011	−0.149*
	(0.056)	(0.063)	(0.066)
t0_ethnicity	−0.112	−0.232	0.385
	(0.377)	(0.368)	(0.351)
t0_politicalinterest	−0.105	0.709***	0.033
	(0.093)	(0.117)	(0.132)
t0_genderMale	−0.092	0.600**	0.626**
	(0.181)	(0.215)	(0.230)
t0_outparty_closeness	0.433***	0.027	0.026
	(0.053)	(0.051)	(0.060)
partystrength	−0.474***	−0.299*	−0.693***
	(0.112)	(0.118)	(0.149)
TreatmentTreatment:convo_topicRedistribution	−0.826*	−0.445	−0.381
	(0.350)	(0.404)	(0.412)
R^2	0.295	0.161	0.087
Adj. R^2	0.283	0.148	0.073
Num. obs.	575	574	574
RMSE	1.983	2.397	2.505
N Clusters	297	297	297

$^{***}p < 0.001$; $^{**}p < 0.01$; $^{*}p < 0.05$
