# Supplementary material for: Cross-partisan discussions reduced political polarization between UK voters, but less so when they disagreed
Source: Commun Psychol. 2024 Jan 5;2:5. doi: 10.1038/s44271-023-00051-8 (PMC11332051; doi:10.1038/s44271-023-00051-8)
Supplement: Supplementary file 3 — Reporting Summary [file 44271_2023_51_MOESM3_ESM.pdf]

nature portfolio

Corresponding author(s): DBPR - COMMSPSYCHOL-23-0198-T

Last updated by author(s): Aug 22, 2023

Reporting Summary

Nature Portfolio wishes to improve the reproducibility of the work that we publish. This form provides structure for consistency and transparency in reporting. For further information on Nature Portfolio policies, see our Editorial Policies and the Editorial Policy Checklist.

Statistics

For all statistical analyses, confirm that the following items are present in the figure legend, table legend, main text, or Methods section.

n/a	Confirmed	
☐	☒	The exact sample size (n) for each experimental group/condition, given as a discrete number and unit of measurement
☐	☒	A statement on whether measurements were taken from distinct samples or whether the same sample was measured repeatedly
☐	☒	The statistical test(s) used AND whether they are one- or two-sided<br>*Only common tests should be described solely by name; describe more complex techniques in the Methods section.*
☐	☒	A description of all covariates tested
☐	☒	A description of any assumptions or corrections, such as tests of normality and adjustment for multiple comparisons
☐	☒	A full description of the statistical parameters including central tendency (e.g. means) or other basic estimates (e.g. regression coefficient) AND variation (e.g. standard deviation) or associated estimates of uncertainty (e.g. confidence intervals)
☐	☒	For null hypothesis testing, the test statistic (e.g. F, t, r) with confidence intervals, effect sizes, degrees of freedom and P value noted<br>*Give P values as exact values whenever suitable.*
☒	☐	For Bayesian analysis, information on the choice of priors and Markov chain Monte Carlo settings
☒	☐	For hierarchical and complex designs, identification of the appropriate level for tests and full reporting of outcomes
☐	☒	Estimates of effect sizes (e.g. Cohen's d, Pearson's r), indicating how they were calculated

Our web collection on statistics for biologists contains articles on many of the points above.

Software and code

Policy information about availability of computer code

Data collection	The data was collected with the use of Qualtrics (pre-screener and follow-up questionnaire) and the custom-built platform (experiment)
Data analysis	The data analysis was conducted with the use of R, version 4.3.1.

For manuscripts utilizing custom algorithms or software that are central to the research but not yet described in published literature, software must be made available to editors and reviewers. We strongly encourage code deposition in a community repository (e.g. GitHub). See the Nature Portfolio guidelines for submitting code & software for further information.

Data

Policy information about availability of data

All manuscripts must include a data availability statement. This statement should provide the following information, where applicable:

- Accession codes, unique identifiers, or web links for publicly available datasets
- A description of any restrictions on data availability
- For clinical datasets or third party data, please ensure that the statement adheres to our policy

Anonymized data can be accessed here: https://osf.io/q4hjd/

Human research participants

Policy information about studies involving human research participants and Sex and Gender in Research.

Reporting on sex and gender	I do not conduct analyses based on sex or gender.
Population characteristics	Appendix B.3 shows demographic information of the entire sample. Respondents had a mean age of 44, 57% self-identified as women and 55% of respondents had at least a bachelor's degree.
Recruitment	Recruitment details are in Appendix B1. I recruited participants through Prolific, a research company. Prolific has several features that makes it useful for conducting studies that require following up with participants, such as extensive pre-screening options, direct communication with respondents and a large and active UK user base. I surveyed participants three times: a few days before the experiment, right after the experiment and two-three weeks after the experiment. Participants took the pre-screener and follow-up survey in Qualtrics, but answered pre and post-discussion questions on the Civinc platform where they also had the discussion. I recruited them in nine waves, on nine different days in June 2022.<br><br>A total of 2208 people took the pre-screener. The survey asked several questions on demographics and politics, and took an average of three minutes to complete. Based on answers to the first wave of the pre-screener, I selected two statements out of five that were to be the discussion topics: immigration and redistribution. Selection was based on highest average disagreement between partisans in the first wave. I limited eligibility for the discussion experiment to those who voted Labour or Conservative in the last election, have the UK nationality, have command of the English language and are willing to return for 'a brief, anonymous discussion on a novel chat platform'. This left 1523 respondents. Each respondent was asked to provide a time slot on which they could return for the experiment. Some did not do this, and I randomly dropped mostly Labour voters to invite a balanced sample of Labour and Conservative voters to come to the platform in each wave. A total of 1090 respondents were invited to take part in the experiment. I sent the invitation through a private message in Prolific's internal messaging system. Invitees were asked to mark the time slot in their agenda. They were also instructed that participation and payment were conditional upon arriving exactly on time. A few hours before each time slot, I make the study available on Prolific to invited participants. Thirty minutes before each time slot, I sent participants a final message reminding them that the study was about to start.<br>777 participants, around 60% of those invited, showed up. An algorithm immediately randomly assigned them to the control condition or the treatment condition. About 50 were excluded because they were too late or too early to be matched. Others were excluded, but paid, because they could not find a match. This was due to an imbalance in partisanship within waves. Though invitations to each wave were balanced by partisanship, attendance was not always. As instructed, 78 participants reported to me by private message that they could not find a match, after having waited for at least five minutes. The data show how long participants spend on the survey, and whether they enter the matching process, allowing me to see whether they actually waited five minutes. Some participants did not contact me, but instead re-entered the platform. In this case, I took out both these participants and their discussion partner. A few others reported a technical glitch: the platform froze, or they were thrown out. If this happened before the treatment, I took them out. If it happened during the treatment, I also took out their discussion partner. I left them in if it happened after the treatment. Supplementary Fig. 2 shows that only education levels are predictive of whether participants made it into the final sample or not. Those in the final sample are more likely to have a master's degree and those who are not in the final sample are more likely to have only higher secondary education. Other than this, there is a balance on treatment assignment as well as policy attitudes, closeness towards parties, and ideology in the pre-screener.<br><br>Results are thus limited to participants who indicated to be willing to have a brief, anonymous discussion on a novel chat platform, showed up for the study, and completed the discussion.
Ethics oversight	Ethics Committee of the European University Institute

Note that full information on the approval of the study protocol must also be provided in the manuscript.

Field-specific reporting

Please select the one below that is the best fit for your research. If you are not sure, read the appropriate sections before making your selection.

☐ Life sciences ☒ Behavioural & social sciences ☐ Ecological, evolutionary & environmental sciences

For a reference copy of the document with all sections, see nature.com/documents/nr-reporting-summary-flat.pdf

Behavioural & social sciences study design

All studies must disclose on these points even when the disclosure is negative.

Study description	The randomized online experiment matched respondents in the treatment condition to a political opposite, for a 10-minute chat-based discussion on either redistribution or immigration. I asked a series of outcome measures related to political polarization after the discussion. Responses to these measures were compared to those given by other respondents, who were randomly assigned to a wait-list control group, and then also had a chat discussion.
Research sample	The sample consists of 582 UK Labour and Conservative voters. The sample is higher-educated and younger than the UK population (average age of 44, 57% women more than 50% with at least a bachelor's degree). I had several requirements for respondent selection (see Appendix B for more details). The study's interest was in the effect of cross-partisan discussions. Thus, respondents had to be Labour or Conservative voters. They had to be willing to have a discussion on a chat platform, and return to the actual platform on a pre-specified timeslot.
Sampling strategy	I limited the pre-screener survey to Labour and Conservative voters, UK nationals and those with command of the English language. For those willing to return for the experiment, I stratified by partisanship. I invited a balanced sample of 50/50 Labour/Conservative supporters on to the platform, in waves. I pre-registered a sample size of N = 600 (https://osf.io/q4hjd/), based on a power calculation with the pwr package in R. I based expected effect size on recent studies that also tested effects of cross-partisan discussions (e.g. Broockman and Santoro, 2022; Rossiter, 2023).
Data collection	I recruited participants using Prolific, an online crowdwork platform. The pre-screener survey, and the follow-up survey were conducted in Qualtrics. The experiment took place on the discussion platform developed by Civinc. The pre-discussion outcome measures for the control group, and post-discussion measures for both groups, were asked on this platform as well. Nobody was present except participants and the researcher. The researcher was blind to the experimental condition during data collection (respondents were assigned to treatment or control by an algorithm on the platform, and only after the experiment could I see in the data which condition respondents had been assigned to).
Timing	Start pre-screener: June 10, 2022. Start experiment: June 11, 2022. End experiment: June 21, 2022. Follow-up survey: June 29 - July 6 2022.
Data exclusions	Exclusion criteria were pre-registered (https://osf.io/q4hjd/), and more details are in Appendix B. Participants would be excluded when they did not want to participate in the study, did not vote Labour or Conservative, were randomly dropped for partisan balance on the platform, did not show up to participate and showed up too late or early so that they could not find a match. 2255 people took the pre-screener, 1090 were invited to take part in the experiment. 777 showed up. 650 were on time and found a match. I excluded another 42 participants because they entered twice, because their partner entered twice or because they ran into a technical error, or their partner did. 28 participants may have attritted. 15 left the discussions without contacting me (which they had been instructed to do in case anything went wrong), and 12 were not matched, without contacting me. It is unlikely that all 28 of them attritted, but I do run tests to see whether attrition is predicted by covariates or treatment assignment (it is not).
Non-participation	See above for details on this.
Randomization	Participants were randomly assigned to a control or a treatment group, by an algorithm on the platform. Table 2 in the appendix shows that randomization was successful.

Reporting for specific materials, systems and methods

We require information from authors about some types of materials, experimental systems and methods used in many studies. Here, indicate whether each material, system or method listed is relevant to your study. If you are not sure if a list item applies to your research, read the appropriate section before selecting a response.

Materials & experimental systems

n/a	Involved in the study	
☒	☐	Antibodies
☒	☐	Eukaryotic cell lines
☒	☐	Palaeontology and archaeology
☒	☐	Animals and other organisms
☒	☐	Clinical data
☒	☐	Dual use research of concern

Methods

n/a	Involved in the study	
☒	☐	ChIP-seq
☒	☐	Flow cytometry
☒	☐	MRI-based neuroimaging
